# Rousing Homeopathic Demonstration

**Published:** 1892-05

**Authors:** 


### ROUSING HOMEOPATHIC DEMONSTRATION.

GALVESTON, May 10.—The State Association of homeopaths which had been called to hold its annual session here to-day, failed to materialize. There were only three delegates from the interior present. These, with one local delegate, met in the parlor of the Beach Hotel and looked at each other and smiled [must have been a cheerful smile.—ED.] as much as to say "What are we here for?" They adjourned to meet on 11th June. —Telegram to *Statesman.*

The Bi-Chloride-Whisky-Cure-Annex did it; swamped their little boat. We told you so; clean case of tail wag the dog. No doubt many will now follow the Apostle Bragg's example and catch on to the whisky cure for a living.

Seriously, there are some good and intelligent men in the homeopathic ranks who are not homeopaths; who do not hesitate to say they do not believe or practice any of the Hahnemannian absurdities, but use any and all *rational* means of cure. As rational, however, they still stick to the alleged law of *similia.* If they would discard this, and drop the name, there would be no difference between them and *physicians*, and they would receive recognition as physicians and be treated with proper respect by the medical profession. The homeopaths, so-called, are perhaps well meaning enough (we mean the honest ones) but they are deluded. Those who see through the delusion and still call themselves homeopaths are sailing under false colors. They lay themselves liable to be suspected of using the name as a trade mark to catch practice. Why not be honest and drop the name and say "I am a *physician* and recognize no *pathy*."

